# Disparities in structural brain imaging in older adults from rural communities in Southern Nevada

**DOI:** 10.3389/fnagi.2024.1465744

**Published:** 2024-10-04

**Authors:** Xiaowei Zhuang, Dietmar Cordes, Jessica Z. K. Caldwell, Andrew R. Bender, Justin B. Miller

**Affiliations:** ^1^Cleveland Clinic Lou Ruvo Center for Brain Health, Las Vegas, NV, United States; ^2^Interdisciplinary Neuroscience PhD Program, University of Nevada, Las Vegas, Las Vegas, NV, United States; ^3^Institute of Cognitive Science, University of Colorado Boulder, Boulder, CO, United States

**Keywords:** rural–urban differences, cortical thickness, neighborhood disadvantage, rural–urban commuting area, dementia, Alzheimer’s disease

## Abstract

**Introduction:**

Identifying the associations between rural-living or neighborhood disadvantage and neurobiology may clarify rural–urban disparities in older adults with cognitive impairment related to Alzheimer’s disease.

**Methods:**

We examined rural–urban differences and neighborhood disadvantages in brain cortical thickness (CT) measures among 71 rural and 87 urban-dwelling older adults. Analysis of covariance was used to test each FreeSurfer-derived CT measures’ associations with rural–urban living, clinical impairment status, and their interactions. Post-hoc linear regressions were used to test the association between CT measures and neighborhood disadvantage index.

**Results:**

Rural-dwelling older adults had thinner cortices in temporal and inferior frontal regions compared to urban participants, especially among clinically normal participants, where the thinner temporal cortex further correlated with higher neighborhood disadvantage. Conversely, rural participants had thicker cortices in superior frontal, parietal and occipital regions.

**Discussion:**

Our results suggest a complex interplay between community contexts and neurobiology. For memory-related regions, rural-living and neighborhood disadvantage might be negatively associated with subjects’ brain structures.

## 1 Introduction

Older adults living in rural communities and disadvantaged neighborhoods are at a heightened risk for Alzheimer’s Disease (AD) than those living in urban areas and advantaged neighborhoods (Majoka and Schimming, 2021; Rahman et al., 2021; Liu et al., 2022; Wiese et al., 2023). This risk difference parallels other place-based disparities in health behaviors and mortalities, and is rooted in the neighborhood demographic, social, cultural, economic, and physical conditions in which human beings live and age (Meilleur et al., 2013; Aggarwal et al., 2021; Cross et al., 2021; Turecamo et al., 2023). Given the fact that rural areas often have a higher proportion of elderly individuals that are under-represented in AD-related studies, there is an emerging demand to include participants from rural and less advantaged neighborhoods in AD research to understand the factors contributing to these disparities and more inclusively promote healthy aging.

Cognitive decline is one of the major clinical symptoms in AD, and neurobiological changes precede and contribute to these clinical impairments (Aisen et al., 2017; Jack et al., 2018). Various socio-demographic, socio-cultural and socio-economic factors may shape residents’ cognition and neurobiology (Aisen et al., 2017; Majoka and Schimming, 2021). Currently, how exactly these factors impact cognitive functioning and brain structures in AD remains unclear. Recent studies investigate such factors individually, in terms of sex-gender, education and socio-economic status (Stern, 2012; Hill et al., 2015; Caldwell et al., 2017, 2019; Cieri et al., 2022). Compared to these individual factors, integrative measures such as residency status and neighborhood disadvantage, offer an opportunity to comprehensively study the complex interplay among the above factors and the cognitive and neurobiological changes relevant to AD.

Rural living in early life could be a risk factor for lower levels of cognitive functioning (Herd et al., 2021). Better verbal memory performances have also been reported in rural-dwelling older adults (Miller et al., 2023). Investigating neurobiological changes associated with neighborhood contexts might help elucidate these mixed findings. To this end, neighborhood disadvantage has been associated with AD-specific patterns of neurodegeneration such as hippocampal volume loss (Hunt et al., 2020, 2021) and AD-related neuropathological changes (Powell et al., 2020) across lifespan. To date, these studies have been primarily conducted in clinically normal adults living in urban communities. It remains an open question how rural residency or disadvantaged neighborhood will be associated with AD biomarkers, such as brain regional structures, in both cognitively normal and impaired older adults.

In an effort to bridge this gap, we explore (1) rural–urban differences in brain structures and (2) the association between neighborhood disadvantage and these brain structures in both clinically normal and impaired participants. We particularly seek to examine the association of rural residency or neighborhood disadvantage with brain cortical thickness (CT) measures, hypothesizing that living in rural or a more disadvantaged area could be associated with thinner cortex in regions related to AD.

## 2 Materials and methods

### 2.1 Participants

Data for this project were drawn from participants enrolled in the Nevada Exploratory Alzheimer’s Disease Research Center (NVeADRC)^1^ and the Nevada Center for Neurodegeneration and Translational Neuroscience (CNTN).^2^ Both studies have been reviewed and approved locally by the Cleveland Clinic Institutional Review Board and all participants gave written, informed consent prior to participation.

Details of these cohorts have been previously reported (Ritter et al., 2018; Sabbagh et al., 2021; Miller et al., 2023). Briefly, the NVeADRC is actively enrolling community-dwelling adults over the age of 50 that maintain a primary and current residency in a non-metropolitan area surrounding Las Vegas, NV. CNTN is a longitudinal, natural history study that is actively enrolling the clinical population at the Cleveland Clinic Nevada in Las Vegas. In both studies, following the aligned protocols, annual visits were conducted at the same single site, including a clinical examination, neuropsychological assessment based on the Uniform Data Set (v3) (Weintraub et al., 2018), and brain magnetic resonance imaging (MRI) acquisition. Demographic information including age, sex, years of education (YOE), race and ethnicity were self-reported and collected during each clinical visit.

#### 2.1.1 Rural–urban status and neighborhood disadvantage

Based on participants’ primary and current addresses, Rural–urban commuting area (RUCA) code (USDA ERS – Rural-Urban Commuting Area Codes, 2010) was utilized to characterize their residency status and area deprivation index (ADI) state decile (i.e., ranking within Nevada) (Kind and Buckingham, 2018; University of Wisconsin School of Medicine and Public Health, 2023) was used to characterize their neighborhood disadvantage. Details about RUCA and ADI were included in Supplementary method 1.

Briefly, a higher ADI indicates a more disadvantaged neighborhood and is linked to various negative health outcomes (Mora et al., 2021; Rangachari et al., 2022). In addition, following the guidance from the Health Resources and Services Administration (HRSA) (Health Resources and Services Administration, 2024), NVeADRC participants whose address was associated with RUCA ≥4 were included as the rural cohort; and CNTN participants with RUCA<4 were included as the urban cohort.

#### 2.1.2 Demographic and clinical characteristics

Demographic variables, RUCA, ADI state decile, and clinical diagnoses [cognitively normal, mild cognitive impairment (MCI) or dementia] were obtained for each participant. Given the limited number of individuals with dementia, we collapsed those with MCI and dementia into a unified clinically impaired group. The Montreal Cognitive Assessment (MoCA) (Nasreddine et al., 2005), Clinical Dementia Rating (CDR) (Hughes et al., 1982) sum of boxes, and Rey Auditory Verbal Learning Test (RAVLT) (Schmidt, 1996) immediate (sum of trial1-5) and delayed-recall scores were used to assess overall cognitive and memory function.

#### 2.1.3 Structural MRI

MRI scans were collected locally for both studies on a Siemens 3 T scanner with a standard MPRAGE sequence at the same visit as the clinical examination. Details of MRI acquisitions and processes (Fischl, 2012) were included in Supplementary method 2.

Our analyses focused on 68 regional CT measures from subject-specific whole-brain anatomical labeling (Desikan et al., 2006) and 2 average CT measures of the two hemispheres, without any a prior selection.

### 2.2 Statistical analysis

All statistical analyses were conducted in matrix laboratory (MATLAB) 2022b.^3^ Confidence intervals for effect-sizes were computed using the MBESS package in R (Kelley, 2007). If not otherwise stated, statistical significance levels were established at uncorrected *p* ≤ 0.05.

#### 2.2.1 Demographic comparison

Differences between rural and urban participants were explored using a two-sample t-test for continuous variables and a Chi-square test for categorical variables.

#### 2.2.2 Analysis of covariance (ANCOVA)

Our primary analysis was to investigate rural–urban differences of each CT measure and examine whether these differences would differ between clinically normal and impaired stages. To this end, an ANCOVA with main effects of residency (rural vs. urban) and clinical impairment status (normal vs. impaired), along with their interactions was used. Age, sex, and YOE were included as covariates.

Since ANCOVA was conducted on each of the 70 CT measures, uncorrected *p*-values were corrected for 70×3 comparisons for both main and interaction effects using the false discovery rate (FDR) method. Statistical significance levels were established at *p_FDR_* ≤ 0.05. For the following post-hoc analyses, each CT measure was adjusted for sex, age, and YOE using coefficients estimated in the ANCOVA.

#### 2.2.3 Post-hoc effect-sizes

Cohen’s d (d) between rural and urban groups (i.e., rural–urban) of CT measures were computed for the overall samples, and for clinically normal and impaired participants, respectively. Effect-sizes between normal and impaired groups (i.e., normal-impaired) were calculated to approximate the relative degree of CT differences between different clinical stages in rural cohorts relative to urban cohorts.

#### 2.2.4 Post-hoc association between CT and ADI

For CT measures with a significant interaction effect, we performed a linear regression analysis to determine whether there was an association between neighborhood disadvantage and CT measures in clinically normal and impaired cohorts. CT measures adjusting for covariates were used as the dependent variable, and ADI state decile was considered as the independent variable. We also tested whether the slope obtained through linear regression model between CT measures and ADI would differ between clinically normal and impaired groups. Due to the relatively limited sample-sizes, this association analyses were conducted in the post-hoc manner; and given the cohort relevance to AD, we extended this post-hoc association analyses to all temporal regions.

## 3 Results

### 3.1 Participants’ demographics

Participants’ demographic information is summarized in Table 1. Briefly, we assessed 71 rural-dwelling (62.0% women, 71.13 ± 6.45 years old, primarily non-Hispanic (88.5%) White (93.1%), 56.3% clinically normal) and 87 urban-dwelling (43.7% women, 72.16 ± 6.88 years old, primarily non-Hispanic (97.6%) White (88.7%), 52.1% clinically normal) older adults. The ADI state decile was significantly higher in rural than urban cohort, with distributions skewed toward 3–10 in the rural and 1–2 in the urban cohorts, respectively (Supplementary Figure 1).

**Table 1:** Demographics of all participants (big column 1), clinically normal participants (big column 2) and clinically impaired participants (big column 3).

Characteristic	Overall			Clinically normal			Clinically impaired		
	Urban	Rural	Urban–rural differences (*p*-values)	Urban	Rural	Urban–rural differences (*p*-values)	Urban	Rural	Urban–rural differences (*p*-values)
	Mean (SD)	Mean (SD)		Mean (SD)	Mean (SD)		Mean (SD)	Mean (SD)	
	*n* = 87	*n* = 71		*n* = 49	*n* = 37		*n* = 38	*n* = 34	
Sex [No. (%)]			0.02			0.07			
Women	38 (43.7%)	44 (62.0%)		25 (51.0%)	26 (70.3%)		13 (34.2%)	18 (52.9%)	
Men	49 (56.3%)	27 (38.0%)		24 (49.0%)	11 (29.7%)		25 (65.8%)	16 (47.1%)	
Age	72.16 (6.88)	71.13 (6.45)		70.57 (6.96)	69.33 (6.04)		74.21 (6.28)	73.10 (6.39)	
Education in years	16.05 (2.43)	15.59 (2.35)		16.10 (2.46)	16.03 (2.34)		15.97 (2.42)	15.12 (2.31)	
ADI state decile	3.32 (2.10)	5.75 (2.51)	<0.001	3.35 (2.20)	5.54 (2.38)	<0.001	3.29 (2.00)	6.00 (2.68)	<0.001
RUCA code	1.01 (0.11)	4.27 (0.92)	<0.001	1.02 (0.14)	4.28 (1.05)	<0.001	1.00 (0.00)	4.26 (0.76)	<0.001
Ethnicity [No. (%)]			0.01			0.08			0.07
Hispanic	10 (11.5%)	1 (1.4%)		4 (8.1%)	0 (0.0%)		6 (15.8%)	1 (2.9%)	
Non-Hispanic	77 (88.5%)	70 (98.6%)		45 (91.9%)	37 (100.0%)		32 (84.2%)	33 (97.1%)	
Race [No. (%)]									
American Indian or Alaska Native	0 (0.0%)	2 (0.0%)		0 (0.0%)	1 (2.7%)		0 (0.0%)	1 (2.9%)	
Asian	4 (4.6%)	2 (2.8%)		3 (6.1%)	2 (5.4%)		1 (2.6%)	0 (0.0%)	
Black	2 (2.3%)	4 (5.6%)		2 (4.1%)	3 (8.1%)		0 (0.0%)	1 (2.9%)	
Native Hawaiian or Other Pacific Islander	0 (0.0%)	0 (0.0%)		0 (0.0%)	0 (0.0%)		0 (0.0%)	0 (0.0%)	
White	81 (93.1%)	63 (88.7%)		44 (89.8%)	31 (83.8%)		37 (97.4%)	32 (94.1%)	
Other (Specify)	0 (0.0%)	0 (0.0%)		0 (0.0%)	0 (0.0%)		0 (0.0%)	0 (0.0%)	
Clinical Diagnosis [No. (%)]									
Impaired	38 (43.7%)	34 (47.9%)		0 (0.0%)	0 (0.0%)		38 (100.0%)	34 (100.0%)	
Normal	49 (56.3%)	37 (52.1%)		49 (100.0%)	37 (100.0%)		0 (0.0%)	0 (0.0%)	
MoCA	24.36 (3.75)	23.85 (4.05)		26.33 (2.68)	26.3 (2.41)		21.81 (3.39)	21.18 (3.79)	
CDR sum of boxes	1.29 (1.91)	0.89 (1.47)		0.35 (0.86)	0.14 (0.3)		2.51 (2.19)	1.72 (1.77)	
RAVLT immediate	36.94 (10.55)	41.45 (12.57)	0.02	41.82 (7.98)	48.97 (9.97)	<0.001	30.66 (10.19)	33.26 (9.69)	
RAVLT delayed-recall	5.53 (4.36)	7.77 (4.67)	0.002	7.84 (3.60)	10.78 (3.01)	<0.001	2.55 (3.33)	4.50 (3.90)	0.03

In all participants, there were no significant differences in age, YOE, or race (1st column in Table 1). Rural cohort had less Hispanic participants ( $\chi^{2}\left(1\right)$ = 6.14, *p* = 0.01) and more women ( $\chi^{2}\left(1\right)$ = 5.24, *p* = 0.02) than urban cohort. Clinical impairment status, MoCA and CDR sum of boxes scores did not differ between rural and urban participants. However, the rural cohort demonstrated a better memory performance on RAVLT immediate [*t*(156) = 2.45, *p* = 0.02] and delayed recall [*t*(156) = 3.12, *p* = 0.002].

After stratifying by impairment status, there were no significant rural–urban differences for any demographic or cognitive variables expect the RAVLT scores. Rural dwelling order adults still demonstrated better verbal memory performances than urban-dwelling participants (2nd and 3rd column in Table 1).

### 3.2 ANCOVA analyses: structural brain cortical thickness (CT) measures

Table 2 summarizes the residency, clinical impairment and interaction effects in the ANCOVA model for 70 CT measures. Significant FDR-corrected *p*-values (*p*_FDR_ ≤ 0.05) are listed (1st big column). Post-hoc effect-sizes (Cohen’s d) for residency (rural–urban, 2nd big column) and clinical diagnoses (normal-impaired, 3rd big column) are listed for all participants, and for participants in each group, respectively.

**Table 2:** Analysis of covariance (ANCOVA) results for cortical thickness measures.

Brain lobes	Brain regions	Significance level in ANCOVA *p_FDR_*			Cohen’s d: rural–urban (d [95% CI])			Cohen’s d: normal-impaired (d [95% CI])		
		Residency status	Impairment status	Interaction	All participants	Normal participants	Impaired participants	All participants	Rural participants	Urban participants
Temporal	Left Entorhinal		4.96E-02					0.46 [0.14, 0.78]	0.23 [−0.24, 0.70]	0.61 [0.17, 1.04]
	Left Fusiform									
	Left Parahippocampal									
	Left Temporalpole	3.13E-02			−0.50 [−0.82, −0.18]	−0.88 [−1.32, −0.43]	−0.10 [−0.56, 0.37]			
	Left Bankssts	2.83E-03			0.60 [0.28, 0.92]	0.53 [0.09, 0.96]	0.72 [0.24, 1.19]			
	Left Inferiortemporal	3.80E-02			−0.47 [−0.79, −0.15]	−0.63 [−1.07, −0.19]	−0.29 [−0.75, 0.18]			
	Left Middletemporal		1.54E-02					0.56 [0.24, 0.87]	0.27 [−0.20, 0.74]	0.77 [0.33, 1.21]
	Left Superiortemporal		4.13E-02					0.48 [0.16, 0.80]	0.16 [−0.31, 0.62]	0.74 [0.30, 1.18]
	Left Transversetemporal									
Frontal	Left Caudalmiddlefrontal	2.65E-03			0.58 [0.26, 0.90]	0.31 [−0.12, 0.74]	0.95 [0.46, 1.44]			
	Left Lateralorbitofrontal	2.00E-03			−0.66 [−0.98, −0.34]	−0.95 [−1.40, −0.50]	−0.36 [−0.83, 0.11]			
	Left Medialorbitofrontal		4.09E-02					0.49 [0.17, 0.80]	0.15 [−0.32, 0.62]	0.80 [0.35, 1.23]
	Left Parsorbitalis									
	Left Parsopercularis			3.26E-02	0.20 [−0.12, 0.51]	−0.20 [−0.63, 0.23]	0.71 [0.23, 1.18]	0.44 [0.12, 0.75]	−0.05 [−0.51, 0.42]	0.83 [0.38, 1.27]
	Left Parstriangularis									
	Left Rostralmiddlefrontal	8.13E-03			0.51 [0.19, 0.83]	0.44 [0.00, 0.87]	0.64 [0.16, 1.11]			
	Left Superiorfrontal									
	Left Frontalpole									
	Left Paracentral									
	Left Precentral									
Parietal	Left Inferiorparietal	1.35E-04			0.76 [0.43, 1.08]	0.79 [0.35, 1.23]	0.76 [0.28, 1.24]			
	Left Postcentral	1.50E-06			0.90 [0.57, 1.23]	0.65 [0.21, 1.09]	1.29 [0.77, 1.79]			
	Left Precuneus	5.54E-05	1.77E-02		0.77 [0.44, 1.09]	0.69 [0.25, 1.13]	0.97 [0.48, 1.46]	0.46 [0.15, 0.78]	0.42 [−0.06, 0.89]	0.63 [0.20, 1.06]
	Left Superiorparietal	9.77E-08			1.06 [0.72, 1.39]	1.12 [0.66, 1.58]	1.00 [0.51, 1.49]			
	Left Supramarginal	8.13E-03			0.51 [0.19, 0.83]	0.29 [−0.14, 0.72]	0.85 [0.37, 1.33]			
Occipital	Left Cuneus	6.91E-04			0.68 [0.36, 1.00]	0.60 [0.16, 1.03]	0.77 [0.29, 1.25]			
	Left Lateraloccipital	1.03E-03			0.66 [0.34, 0.98]	0.67 [0.23, 1.11]	0.67 [0.19, 1.14]			
	Left Lingual	4.09E-02			0.44 [0.12, 0.75]	0.50 [0.06, 0.93]	0.39 [−0.08, 0.85]			
	Left Pericalcarine	7.98E-11	4.40E-02		1.25 [0.91, 1.59]	1.05 [0.59, 1.50]	1.50 [0.97, 2.02]	−0.39 [−0.70, −0.07]	−0.72 [−1.20, −0.23]	−0.10 [−0.53, 0.32]
Cingulate	Left Caudalanteriorcingulate	3.80E-02			−0.46 [−0.78, −0.15]	−0.56 [−1.00, −0.13]	−0.33 [−0.80, 0.14]			
	Left Isthmuscingulate									
	Left Posteriorcingulate									
	Left Rostralanteriorcingulate									
Insula	Left Insula	4.65E-02	4.16E-02	3.24E-02	−0.46 [−0.78, −0.14]	−0.85 [−1.30, −0.40]	0.04 [−0.43, 0.50]	0.49 [0.17, 0.81]	−0.01 [−0.48, 0.45]	0.86 [0.42, 1.30]
Mean	Left Meanthickness	3.13E-02	4.40E-02		0.42 [0.10, 0.74]	0.23 [−0.20, 0.66]	0.71 [0.23, 1.18]	0.44 [0.12, 0.75]	0.20 [−0.27, 0.67]	0.68 [0.24, 1.11]
Temporal	Right Entorhinal		6.77E-03					0.61 [0.28, 0.93]	0.50 [0.03, 0.97]	0.67 [0.23, 1.10]
	Right Fusiform									
	Right Parahippocampal			4.16E-02	−0.30 [−0.61, 0.02]	−0.68 [−1.12, −0.24]	0.17 [−0.29, 0.63]	0.37 [0.06, 0.69]	−0.11 [−0.58, 0.35]	0.69 [0.25, 1.12]
	Right Temporalpole		4.40E-02					0.47 [0.15, 0.79]	0.22 [−0.25, 0.69]	0.65 [0.22, 1.09]
	Right Bankssts									
	Right Inferiortemporal									
	Right Middletemporal		1.18E-03					0.71 [0.39, 1.03]	0.44 [−0.04, 0.90]	0.92 [0.48, 1.37]
	Right Superiortemporal									
	Right Transversetemporal	6.70E-04	3.80E-02		0.65 [0.32, 0.97]	0.51 [0.08, 0.95]	0.87 [0.39, 1.36]	0.43 [0.11, 0.75]	0.35 [−0.12, 0.82]	0.57 [0.14, 1.00]
Frontal	Right Caudalmiddlefrontal	1.34E-03			0.64 [0.32, 0.96]	0.70 [0.26, 1.14]	0.61 [0.14, 1.08]			
	Right Lateralorbitofrontal	8.13E-03		3.80E-02	−0.58 [−0.90, −0.26]	−1.00 [−1.45, −0.54]	−0.11 [−0.57, 0.36]	0.30 [−0.01, 0.62]	−0.20 [−0.66, 0.27]	0.68 [0.24, 1.11]
	Right Medialorbitofrontal									
	Right Parsorbitalis									
	Right Parsopercularis									
	Right Parstriangularis									
	Right Rostralmiddlefrontal	5.92E-04			0.68 [0.36, 1.00]	0.63 [0.19, 1.06]	0.77 [0.29, 1.25]			
	Right Superiorfrontal									
	Right Frontalpole									
	Right Paracentral									
	Right Precentral									
Parietal	Right Inferiorparietal	2.11E-04	4.09E-02		0.70 [0.38, 1.02]	0.60 [0.16, 1.03]	0.89 [0.40, 1.37]	0.42 [0.10, 0.73]	0.28 [−0.19, 0.74]	0.63 [0.19, 1.06]
	Right Postcentral	1.12E-04			0.75 [0.42, 1.07]	0.69 [0.25, 1.12]	0.87 [0.39, 1.36]			
	Right Precuneus	2.45E-06	2.83E-03	3.80E-02	0.82 [0.49, 1.14]	0.49 [0.06, 0.93]	1.38 [0.86, 1.89]	0.57 [0.25, 0.89]	0.20 [−0.27, 0.67]	1.02 [0.57, 1.47]
	Right Superiorparietal	8.96E-08			1.06 [0.72, 1.39]	1.07 [0.61, 1.52]	1.07 [0.57, 1.56]			
	Right Supramarginal	5.26E-03			0.53 [0.21, 0.85]	0.37 [−0.07, 0.79]	0.82 [0.34, 1.30]			
Occipital	Right Cuneus	1.59E-04			0.75 [0.43, 1.08]	0.59 [0.15, 1.02]	0.96 [0.46, 1.44]			
	Right Lateraloccipital	9.73E-04			0.67 [0.34, 0.99]	0.77 [0.32, 1.21]	0.59 [0.11, 1.06]			
	Right Lingual	7.22E-03			0.56 [0.24, 0.88]	0.55 [0.11, 0.98]	0.57 [0.09, 1.04]			
	Right Pericalcarine	1.21E-15			1.59 [1.23, 1.95]	1.42 [0.94, 1.90]	1.81 [1.25, 2.35]			
Cingulate	Right Caudalanteriorcingulate									
	Right Isthmuscingulate									
	Right Posteriorcingulate									
	Right Rostralanteriorcingulate									
Insula	Right Insula		3.13E-02					0.51 [0.19, 0.83]	0.32 [−0.15, 0.79]	0.65 [0.21, 1.08]
Mean	Right Meanthickness	2.83E-03	3.80E-02		0.55 [0.23, 0.87]	0.36 [−0.07, 0.79]	0.86 [0.38, 1.35]	0.44 [0.13, 0.76]	0.21 [−0.25, 0.68]	0.71 [0.27, 1.14]

Significant levels [false discovery rate (FDR) corrected *p*-values (*p_FDR_*)] are listed for residency effect (rural or urban), clinical impairment effect (normal or impaired) and their interaction effect in ANCOVA (big column 1). Post-hoc effect-sizes (Cohen’s d) between rural and urban participants are computed using all, only clinically normal and only clinically impaired participants, respectively (big column 2). A positive effect-size indicates thicker cortex in rural-dwelling than urban-dwelling participants. Post-hoc effect-sizes (Cohen’s d) between normal and impaired participants are calculated using all, only rural and only urban participants (big column 3). A positive effect-size indicates thicker cortex in normal than impaired participants. 95% confidence intervals for each post-hoc effect-size (Cohen’s d) are listed in square brackets. Thickness measures used to calculate the effect-sizes have been adjusted for age, sex and education level using coefficients estimated in the ANCOVA.

#### 3.2.1 Main effect

Our results showed that 32 out of 70 CT measures demonstrated significant residency effects (*p_FDR_* ≤ 0.05, Table 2), and these regions could be divided into two categories.

**Figure 1:**
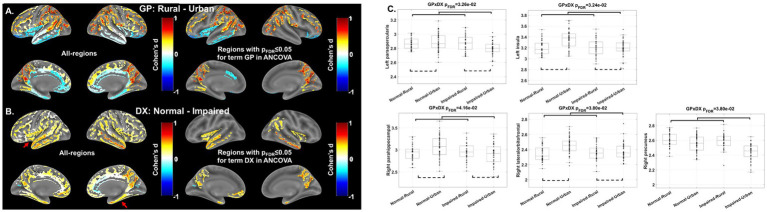
Analysis of covariance (ANCOVA) results on cortical thickness measures. **(A)** Residency effect. Post-hoc effect-sizes (Cohen’s d) between groups (GP: Rural vs. urban) for whole-brain (Left) and regions with significant residency effect (p_FDR_ ≤ 0.05, Right) in the ANCOVA. **(B)** Impairment effect. Post-hoc Cohen’s d of Normal vs. impaired for whole-brain (Left) and regions with significant impairment effect in the ANCOVA (p_FDR_ ≤ 0.05, Right). **(C)** Interaction effect. Five regions showed significant interaction effect between residency and impairment (GPxDX) in the ANCOVA analysis. Thickness measures used in the post-hoc analysis and plotted here have been adjusted for age, sex and education in the ANCOVA model. GP: Residency group (i.e., rural or urban); DX: Diagnosis (i.e., clinical impairment status); FDR: false discovery rate.

First, the inferior frontal and temporal regions demonstrated significantly thinner cortices in rural than urban participants on the post-hoc effect-size (d) maps (blue in Figure 1A). Stratified analyses in clinically normal and impaired participants further showed that these differences were more pronounced in clinically normal than impaired participants (Supplementary Figure 2, left).

In contrast, cortex in parietal, occipital, and superior part of the frontal regions were thicker in rural than urban participants (red in Figure 1A). Stratified analyses suggested that these differences were less predominant in clinically normal than impaired participants (Supplementary Figure 2, right).

For clinical impairment effect, 16 out of 70 CT measures, mainly encompassing temporal and parietal regions, demonstrated a significantly thicker cortex in clinically normal than impaired participants (*p_FDR_* ≤ 0.05, red in Figure 1B, right). A higher CT measure was indeed observed for almost all brain regions in the clinically normal participants (red in Figure 1B, left). Stratified analyses in rural and urban participants further indicated that the clinical impairment effect on CT measures was notably smaller in rural than urban participants (Supplementary Figure 3).

#### 3.2.2 Interaction effect

Significant (*p*_FDR_ ≤ 0.05) interaction effects between impairment and residency on CT measures were found in five brain regions, including left parsopercularis gyrus, left insula, right lateral-orbito-frontal cortex, right parahippocampal gyrus and right precuneus (Figure 1C and Supplementary Figure 4). All five regions except the right precuneus demonstrated thinner cortex in rural than urban participants who were clinically normal. These rural–urban differences became less prominent or even reversed in clinically impaired participants (dashed lines in Figure 1C and Supplementary Figure 4).

### 3.3 Association between neighborhood disadvantage and CT measures

**Figure 2:**
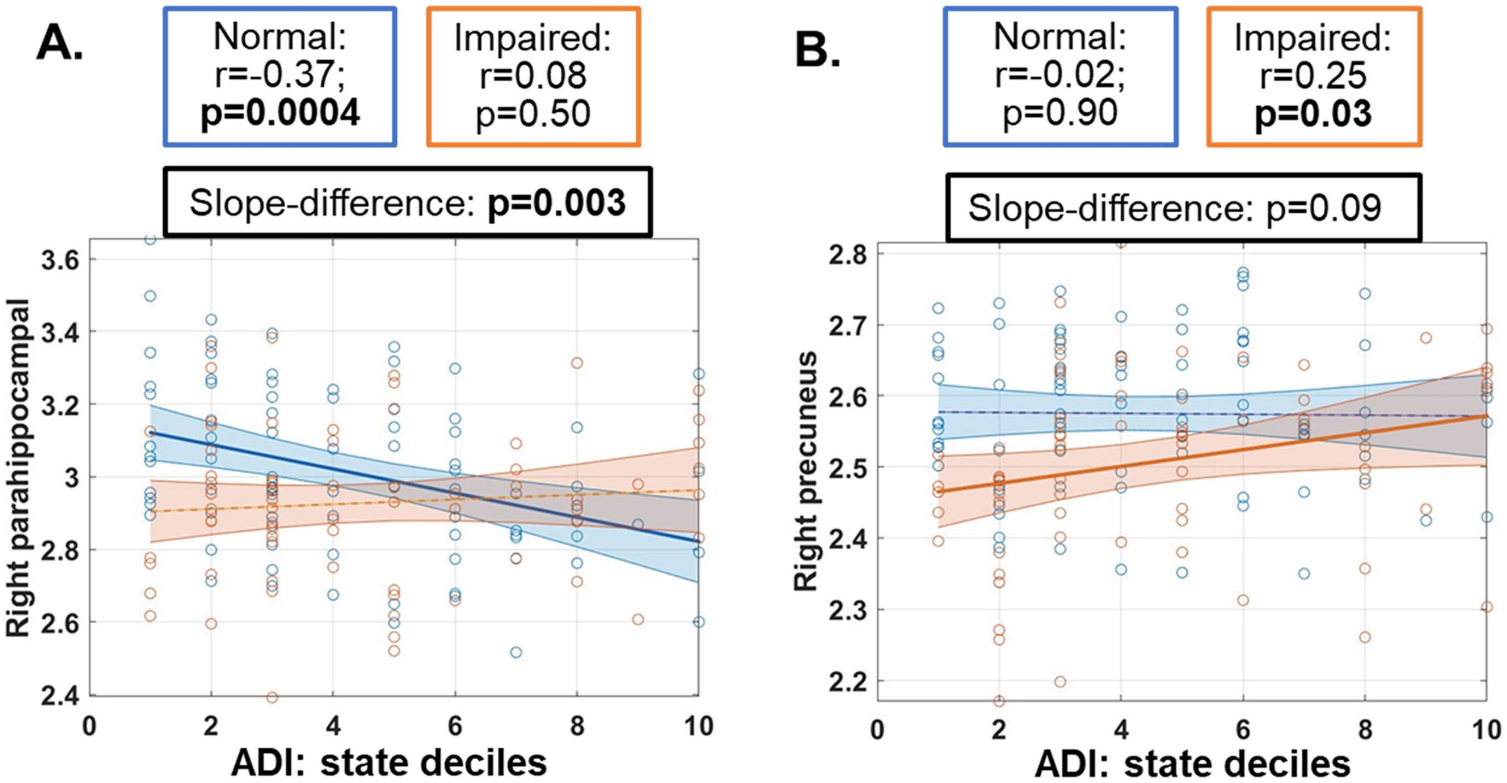
Post-hoc associations between cortical thickness measures and neighborhood area deprivation index (ADI) in both normal (blue) and impaired (orange) participants. ADI ranked within Nevada was used. Regions with significant ANCOVA results were input to this association analyses, and only regions with a significant slope (*p* ≤ 0.05) were plotted here, including parahippocampal gyrus **(A)** and precuneus **(B)**. Cortical thickness measures used in this post-hoc analysis and plotted here have been adjusted for age, sex and education in the ANCOVA model. Partial correlation values (r) with 95% confidence intervals and statistical significance levels (*p*-values) were listed in boxes above the plots.

For the five regions with a significant interaction effect, a significantly negative correlation was observed between the CT of right para-hippocampal gyrus and ADI state decile in normal participants (partial correlation r, [95% confidence intervals (CI)] = −0.37, [−0.54, −0.17], *p* < 0.001, Figure 2A, blue), and this correlation was significantly different from the one in impaired participants (*p* = 0.003, Figure 2A). Similar patterns of associations were observed for all 18 temporal regions (Supplementary Figure 5A), with six regions demonstrating statistical significances (Supplementary Figure 5B).

In contrast, a significant positive correlation between the CT of right precuneus and ADI was observed in impaired participants (*r* = 0.25 [0.01, 0.45], *p* = 0.03, Figure 2B, orange).

## 4 Discussion

Participants from rural areas and less advantaged neighborhoods are at a heightened risk for AD but are underrepresented in AD research (Rahman et al., 2021; Wiese et al., 2023). Our study is among the first to characterize rural–urban differences on brain structural measures and examine the association of neurobiology with neighborhood disadvantage, using both clinically normal and impaired older adults. Our results showed extensive rural–urban disparities on CT measures and significant negative associations between neighborhood disadvantage and CT measures in regions involved in memory and vulnerable to AD.

As we stated in the results section, the observed rural–urban disparities on CT measures could be mainly divided into two categories (summarized in Table 3).

**Table 3:** Summarized residency (rural vs. urban) and clinical impairment (normal vs. impaired) effects for cortical thickness measures.

		All subjects	Normal	Impaired	All subjects	Urban	Rural
Structural MRI-derived cortical thickness measures	Temporal and inferior frontal regions	Rural < Urban (align with our hypothesis)	Rural < Urban (Larger effect size) Negatively associated with higher ADI	Rural < Urban (Smaller effect size)	Normal > Impaired	Normal > Impaired (**Larger** effect size)	Normal > Impaired (**Smaller** effect size)
	Superior frontal, parietal and occipital regions	Rural > urban	Rural > urban (Smaller effect size)	Rural > Urban (Larger effect size)			

### 4.1 First category: temporal and inferior prefrontal regions

Given the putative risk of dementia that rural participants are facing (Wiese et al., 2023), older adults from rural areas exhibiting lower CT measures than their urban peers in these regions (blue in Figure 1A) align with our hypotheses, suggesting disadvantages on brain measures for rural-dwelling older adults.

These rural–urban disparities tend to diminish once clinical impairment onset (red arrows in Supplementary Figure 2), as (1) both rural and urban groups showed a significantly lower CT measures in these regions in impaired participants (red arrows in Figure 1), and (2) these impairment-effect seemed to be less pronounced in rural participants (red arrows in Supplementary Figure 2). These interaction effects between residency and impairment reached statistical significance (*p_FDR_* ≤ 0.05) in three regions from this category (left parsopercularis, right lateral-orbito-frontal cortex, and right parahippocampal gyrus, Figure 1C and Supplementary Figure 4), further consolidating that rural cohort tended to have a less pronounced differences between normal and impaired participants in these regions, as compared to urban participants.

Given the implication of these temporal and frontal regions in memory processes, our findings might imply that rural-dwelling older adults who are cognitively normal may have thinner cortex than their urban peer; and decreases in their CT measures to a lesser degree could notably affect the clinical impairment status, as compared to urban participants.

Additionally for CT measures in this category, a negative association with ADI state deciles was evident in only clinically normal participants (Figure 2A and Supplementary Figure 5), indicating that for cognitively normal individuals, living in a more disadvantaged neighborhood in Nevada was associated with thinner cortex in regions that were mostly involved in memory and vulnerable to AD. These results are consistent with previous reports on ADI associations with thinner cortex in AD signature regions (mostly temporal lobe) and smaller hippocampal volumes in younger unimpaired participants from another US state (Hunt et al., 2020, 2021). Our results additionally suggested that once clinical impairment emerged, impairment effects would be more dominant than the neighborhood effects and masked these associations.

Interestingly, our exploratory analysis with hippocampal volume did not find any association with ADI (Supplementary Figure 6B). Given the vulnerability of hippocampus to aging or AD, and our cohorts were in their 70s [participants in previous studies were in their 60 s (Hunt et al., 2020, 2021)], clinical impairment effect might be again more dominant for these regions and thus masked the neighborhood effect in our analyses.

### 4.2 Second category: parietal, occipital and superior and middle frontal areas

Rural-dwelling participants show thicker cortex in these regions than urban-dwelling participants (red in Figure 1A). Considering the same enhanced dementia risks in rural participants, these rural advantages in brain CT measures were less expected, indicating that a more complex interplay of residency neighborhood contexts with neurobiology may exist. A potential compensatory mechanism might partially explain these observations, as rural participants could recruit other brain regions to supplement the thinner temporal and inferior frontal cortices.

### 4.3 Technical perspectives

In this study, rural–urban status and neighborhood disadvantage index were used in conjunction with separate analyses. We acknowledge that RUCA codes and ADI rankings are to some degree parallel such that more rural areas are likely to be more disadvantaged. However, our interpretation of the underlying components used to derive each metric suggests that they are sufficiently distinct to be used in conjunction. In our analyses, we first evaluated the rural–urban differences in whole-brain CT measures without any prior knowledge and examined the association between CT measures and ADI in a post-hoc manner.

Since ethnicity was significantly different between rural and urban groups, we have repeated our ANCOVA with ethnicity as an additional covariate. All main findings remained the same in this repeated analysis, as revealed by the overall concordance of significance levels in ANCOVA with and without ethnicity as covariates, and the two categories of rural–urban differences on CT measures (Supplementary Figure 7).

### 4.4 Limitation

Several limitations should be considered when interpreting our results.

First, our cross-sectional setting and reliance on single measurement without baseline data could only infer associations between CT measures and residency and/or clinical impairment status. The small sample has further posed a threat to the generalizability of our results. However, we consider our preliminary findings as a starting point to address the underrepresentation of rural-dwelling older adults in neuroimaging research. In addition, our study is specifically designed as a longitudinal study. Future follow-up studies are planned once we have sufficient data available, to evaluate whether a significant cortical thinning exists in urban or rural participants once disease manifests, depending on different regions.

Sex was treated as a covariate in our analyses despite that brain structures could differ between men and women. The relatively small sample sizes have limited our statistical power to stratify our analyses by sex (Supplementary Figure 8). Due to the same reason of limited sample size, the association between ADI and CT measures were also analyzed in a post-hoc manner, focusing on regions with significant interaction effect in ANCOVA. Future analyses with increased sample-sizes might better evaluate the sex-stratified association between neighborhood context and brain structures.

The NVeADRC is a community-dwelling cohort whereas the CNTN recruits urban-dwelling order adults seeking active clinical care. Although these two cohorts did not differ in clinical, cognitive functioning or functional status, the differences in recruitment strategy may bias our findings. However, we observed thinner cortices in memory-related regions in community- and rural-dwelling older adults, which provided confidence to our interpretations that the status of rural-living and neighborhood disadvantage might negatively impact the neurobiology in brain regions involved in memory and vulnerable to AD. Nevertheless, future studies following individuals that have already established care in a rural-based clinical setting could further validate our findings.

In addition, the RUCA code and ADI state deciles in NVeADRC and CNTN were all based on participants’ current and primary residences, thus did not consider participants’ time of settlement. Moreover, besides composite RUCA or ADI scores, other detailed measures such as economic differences and living habits of each participant could also affect their brain structures. In the present study, we chose to focus on the core socio-demographic and socio-economic factors of community, and have not controlled these relevant factors due to the lack of information and the limited sample-size. Future studies evaluating full residency history with updated RUCA codes and detailed economic factors might further consolidate and clarify our results.

More importantly, the average RUCA score in our rural cohort is 4.27, and most participants live 1–2 h away from Las Vegas. Though this RUCA score and this distance confer some disadvantages in receiving healthcare, our cohort can only represent the rural and non-metropolitan areas locally in Nevada, and the disparities experienced by more rural and isolated areas representing by higher RUCA codes are not represented by our cohort. Furthermore, even under similar RUCA codes, detailed rural area characteristics and resource allocations could differ and differentially affect the brain structures of older adults who reside in them. Therefore, additional caution is advised generalizing our results to other rural areas with similar RUCA codes. Nevertheless, our study serves as an important initial step in exploring the impact of community background on brain structures.

## 5 Conclusion

Our cross-sectional study is among the first to characterize rural–urban differences on brain structures and associate CT measures with neighborhood disadvantage in both clinically normal and impaired populations. The results of this observational study demonstrated a complex interplay of rural living and community backgrounds with neurobiological changes in AD. Our findings could potentially aid in designing future studies to more comprehensively understand the rural–urban disparities in AD.

## Data Availability

The datasets used for this study can be found in the Nevada Exploratory Alzheimer’s Disease Research Center (NVeADRC, https://nvadrc.org/) and the Nevada Center for Neurodegeneration and Translational Neuroscience (CNTN, https://nevadacntn.org/), through reasonable requests from qualifying PIs.

## Footnotes

^1^ https://nvadrc.org/

^2^ https://nevadacntn.org/

^3^ https://www.mathworks.com/
