# Supplementary material for: Disparities in structural brain imaging in older adults from rural communities in Southern Nevada
Source: Front Aging Neurosci. 2024 Oct 4;16:1465744. doi: 10.3389/fnagi.2024.1465744 (PMC11486705; doi:10.3389/fnagi.2024.1465744)
Supplement: Supplementary file 1 [file Table_1.DOCX]

**Supplementary Material**

**Disparities in Structural Brain Imaging in Older Adults from Rural Communities in Southern Nevada**

Xiaowei Zhuang^1,2^, Dietmar Cordes^1,3^, Jessica Z.K. Caldwell^1^, Andrew R. Bender^1^ and Justin B. Miller^1^

^1^ Cleveland Clinic Lou Ruvo Center for Brain Health, Las Vegas, NV89106, USA

^2^ Interdisciplinary Neuroscience PhD Program, University of Nevada Las Vegas, Las Vegas, NV89154, USA

^3^ University of Colorado Boulder, Boulder, CO80309, USA

**Correspondence to:**

**Dr. Justin B. Miller:** jbm01@uw.edu

**888 West Bonneville Ave,**

**Las Vegas, NV89106, USA**

**Key words**: Rural-urban differences; Cortical thickness; Neighborhood disadvantage; Rural-Urban Commuting Area (RUCA).

# Supplementary Method 1: Rural-Urban status.

*Rural-urban commuting area (RUCA)*. RUCA code was utilized to characterize participants’ residency status, based on the 5-digit zip code from the participants’ current and primary residency. RUCA is proposed by the United States Department of Agriculture and classifies United States census tracts using measures of population density, urbanization, and daily commuting^1^. It is usually applied to determine the rural urban status of a neighborhood^2^.

*Area deprivation index (ADI).* The ADI measures the overall socioeconomic context of a given census track and includes 17 different factors in its determination, including the theoretical domains of income, education, employment, and housing quality^3,4^. ADI further allows for rankings of neighborhoods by these socioeconomic disadvantages in a region of interest. A higher ADI indicates a more disadvantaged neighborhood and is linked to various negative health outcomes^5,6^. It can be used to inform health delivery and policy, especially for the most disadvantaged neighborhood groups. In our study, ADI state decile (i.e., ranking within Nevada) was used to characterize the neighborhood disadvantage of each participant based on their primary and current addresses.

*Speculated changes in the upcoming updated RUCA categorization*. Current version of RUCA code applied (version 2010) in this study might not be precise considering our data were collected from 2017 to 2023. However, according to the US Department of Agriculture Economic Research Service website, 2020 RUCA code will not be released earlier than Fall 2024. Therefore, we could only speculate potential changes of RUCA categorization in Nevada.

According to the 10th Edition of Nevada Rural and Frontier Health Data Book (Feb. 2021)^7^, from 2009 to 2019, the rural and frontier population in the state of Nevada has increased by 2.6% from 286,028 to 293,594, whereas the urban population in the state of Nevada has increased by 14.8% from 2,711,206 to 3,112,937 (Table 1.3^7^). Based on these numbers, we would speculate that changes in rural and urban populations could further lead to greater rural-urban differences in the 2020 RUCA code, as RUCA code is highly based on population density and urbanization status. Therefore, these changes might be in favor of our findings.

# Supplementary Method 2: Structural MRI collection and process.

For both CNTN and NVeADRC, a high resolution T1-weighted structural MRI was collected locally on a 3T Siemens Skyra scanner with a 32-channel head coil, using the same 3D MPRAGE sequence with the following parameters: TR 2300ms, TE 2.98ms, TI 900ms, flip angle 9 degrees, in-plane resolution 1mmx1mm, and slice thickness 1mm. The MRI data were acquired at the same visit as the clinical examination and cognitive testing.

T1-weighted images were input to the FreeSurfer 6.0^8^ processing pipeline to generate subject-specific anatomical labeling from the Desikan-Killiany atlas^9^, yielding 68 cortical regions. A quality control step was performed using the FreeSurfer’s quality analysis tools (https://surfer.nmr.mgh.harvard.edu/fswiki/QATools) to guarantee only data with reliable cortical reconstruction were included in following analyses. Without any a prior selection, our analyses focused on the 68 regional CT measures and 2 average CT measures of the two hemispheres.

# Supplementary Fig. 1

**Area Deprivation Index (ADI) state decile distributions for the rural-dwelling and urban-dwelling cohorts.**

In our rural-dwelling cohort, the distribution of ADI covered a sufficient range from 3 to 10 whereas in our urban-dwelling cohort, the distribution of ADI was skewed towards 1 and 2. Both distributions were as expected.


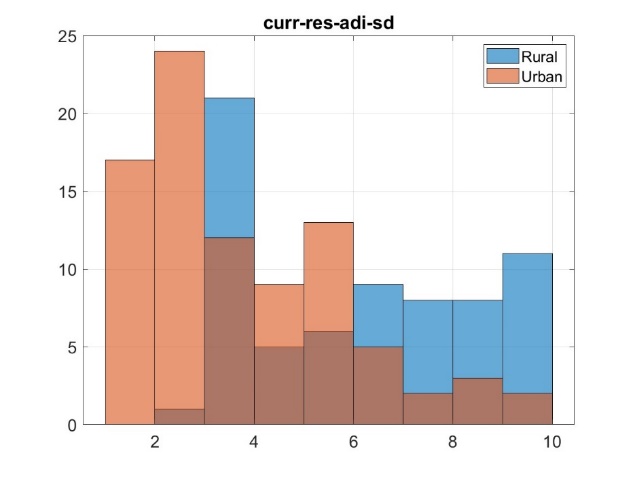


# Supplementary Fig. 2.

**Residency effect in clinically normal (left) and impaired (right) participants**. Post-hoc effect sizes (Cohen’s d) between groups: Rural - Urban for whole-brain (***A***) and regions with significant residency effect ***(****p_FDR_≤0.05,* ***B)*** in the ANCOVA.


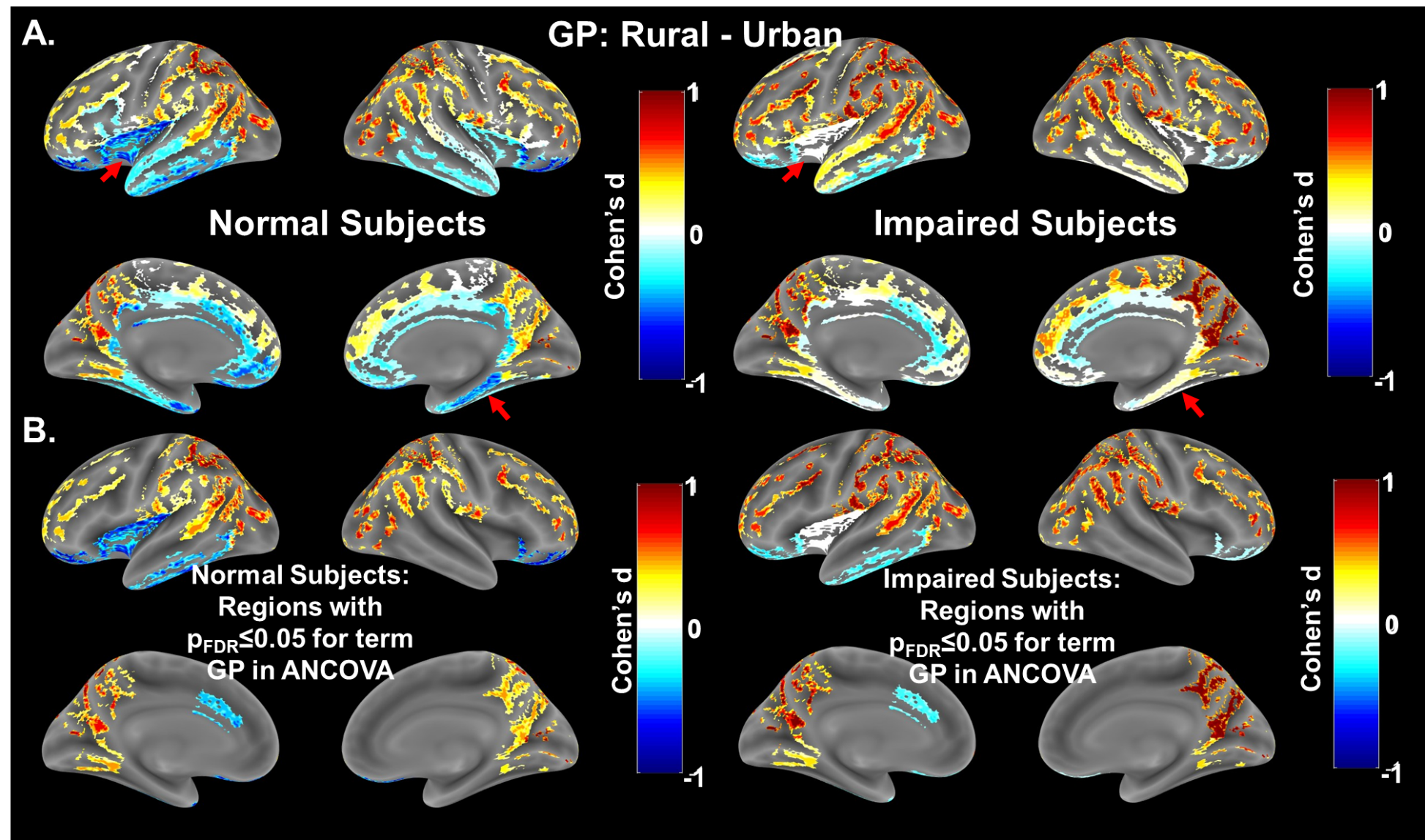


# Supplementary Fig. 3.

**Impairment effect in urban (left) and rural (right) participants**. Post-hoc effect sizes (Cohen’s d) between impairment status: Normal-Impaired for whole-brain (***A***) and regions with significant impairment effect ***(****p_FDR_≤0.05,* ***B)*** in the ANCOVA.


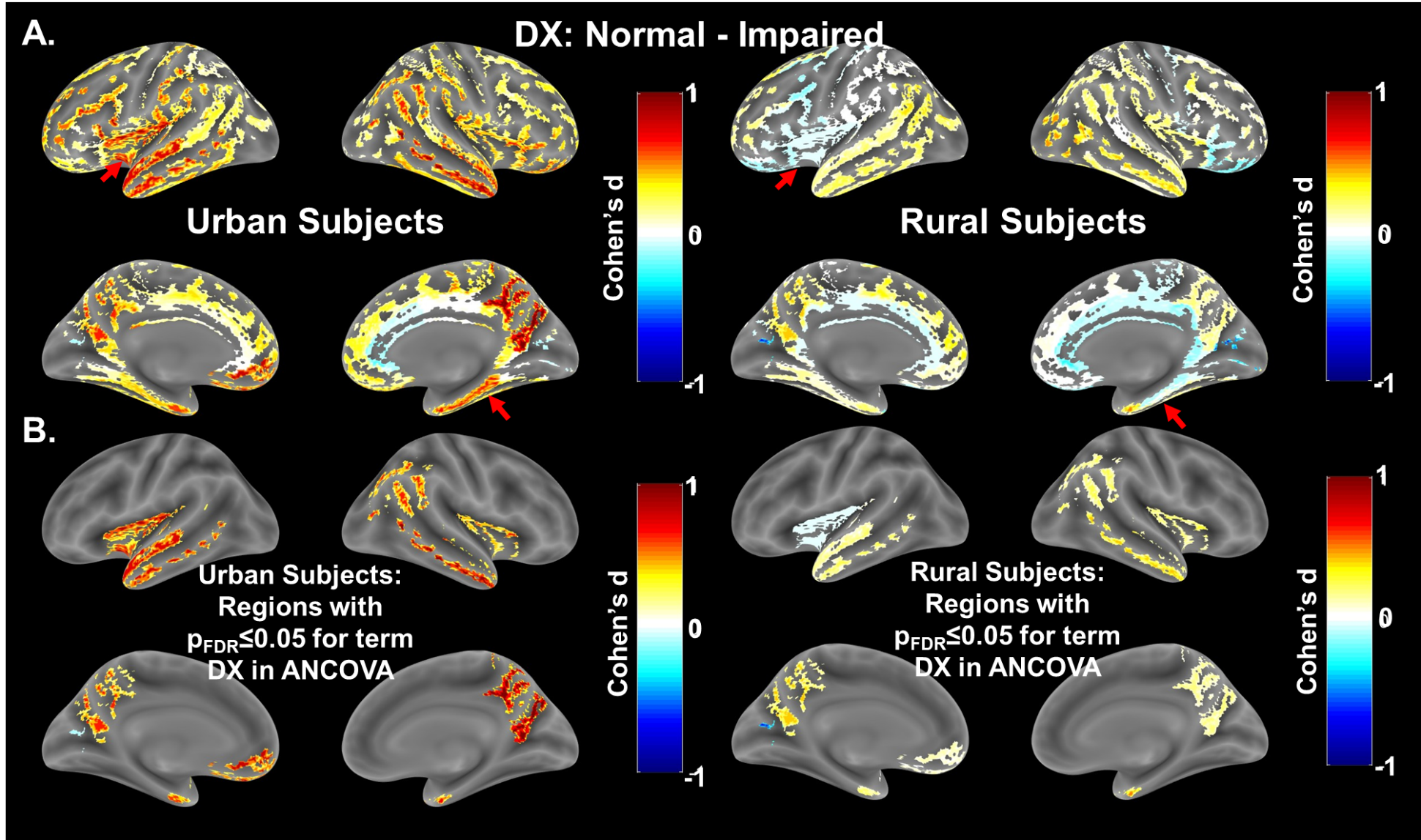


# Supplementary Fig. 4

**Significant interaction effect (*p_FDR_*≤0.05) between residency status (GP, urban-rural) and diagnoses (DX, impaired-normal) in the ANCOVA analysis.** Compared to urban-dwelling participants, rural-dwelling participants shared significantly less differences in cortical thickness (CT) measures between clinically normal and impaired participants. CT values plotted here have been adjusted for age, sex and education. Abbreviations: GP: group; DX: Diagnosis; FDR: false discovery rate.


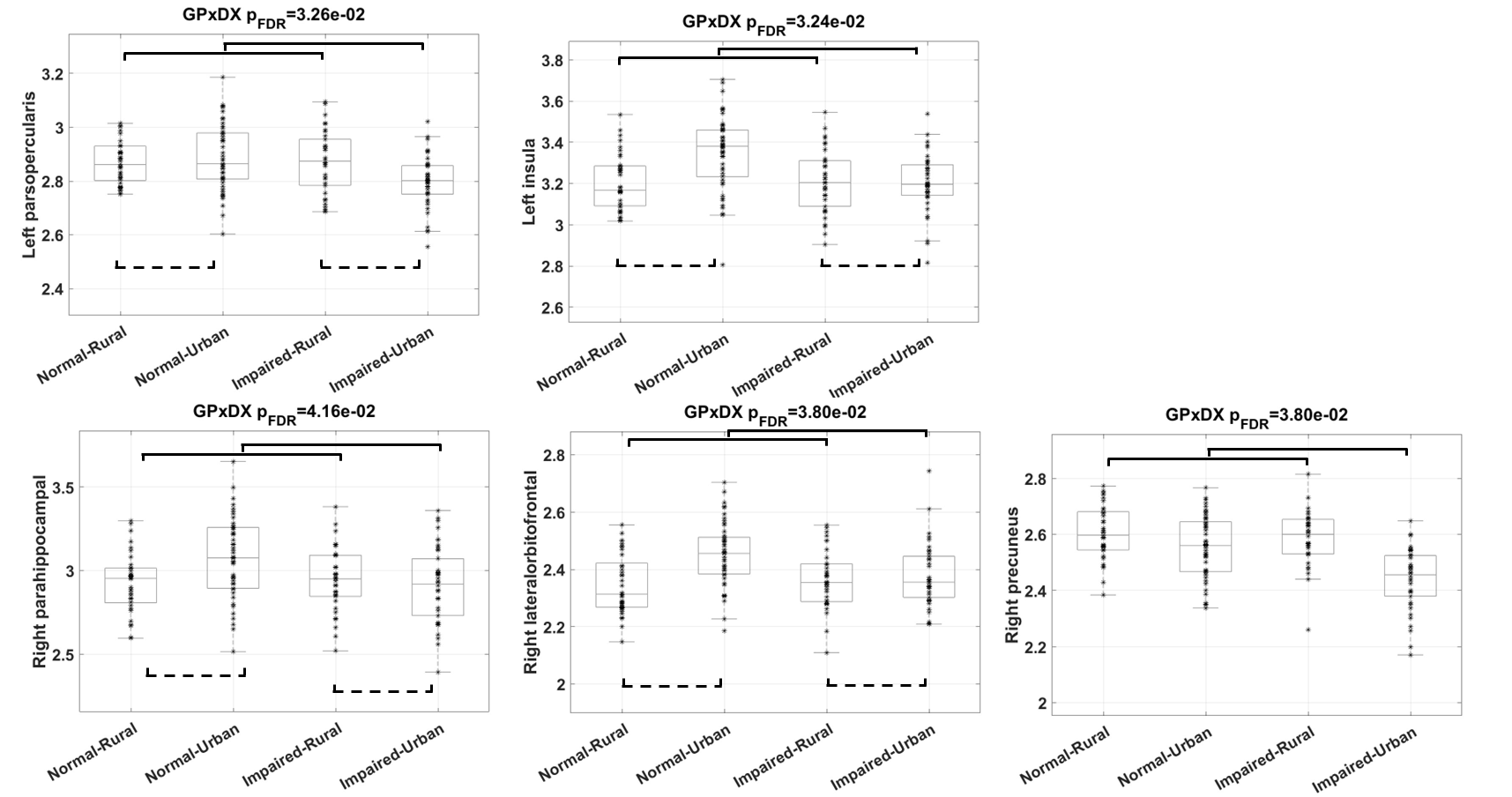


# Supplementary Fig. 5

**Associations between cortical thickness measures and neighborhood area deprivation index (ADI) in both normal (blue in B) and impaired (orange in B) participants.** ADI ranked within Nevada was used. Cortical thickness measures used in this post-hoc analysis and plotted here have been adjusted for age, sex and education in the ANCOVA model.

All temporal regions were input to this association analysis. ***(A).*** Statistical significance *(p-values)* in the association analyses and the partial correlations (r) between ADI and cortical thickness measures for normal and impaired participants were listed. The 95% confidence intervals for each correlation value were listed in square brackets. ***(B).*** Only regions with a significant slope (*p≤0.05*) were plotted.


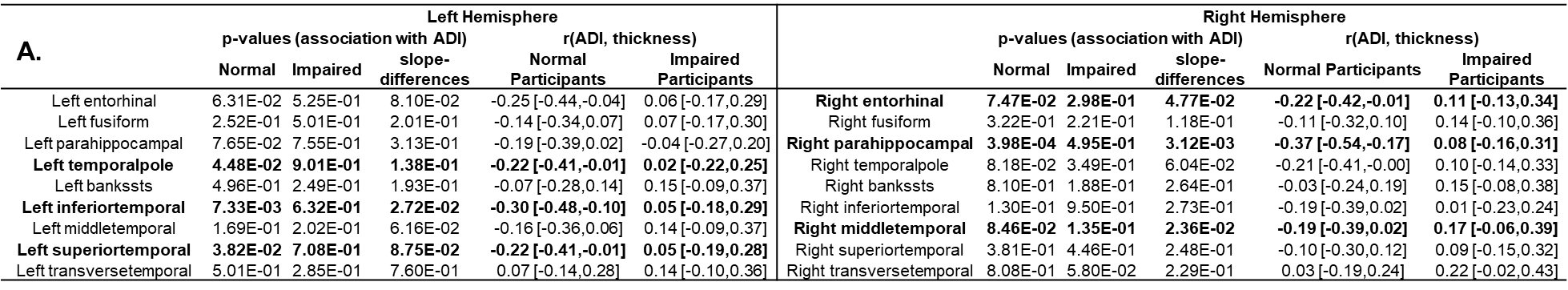

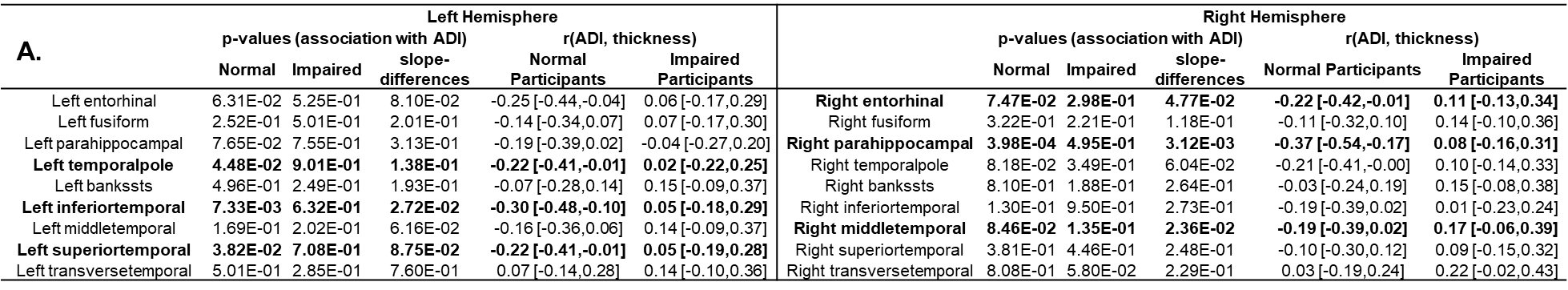


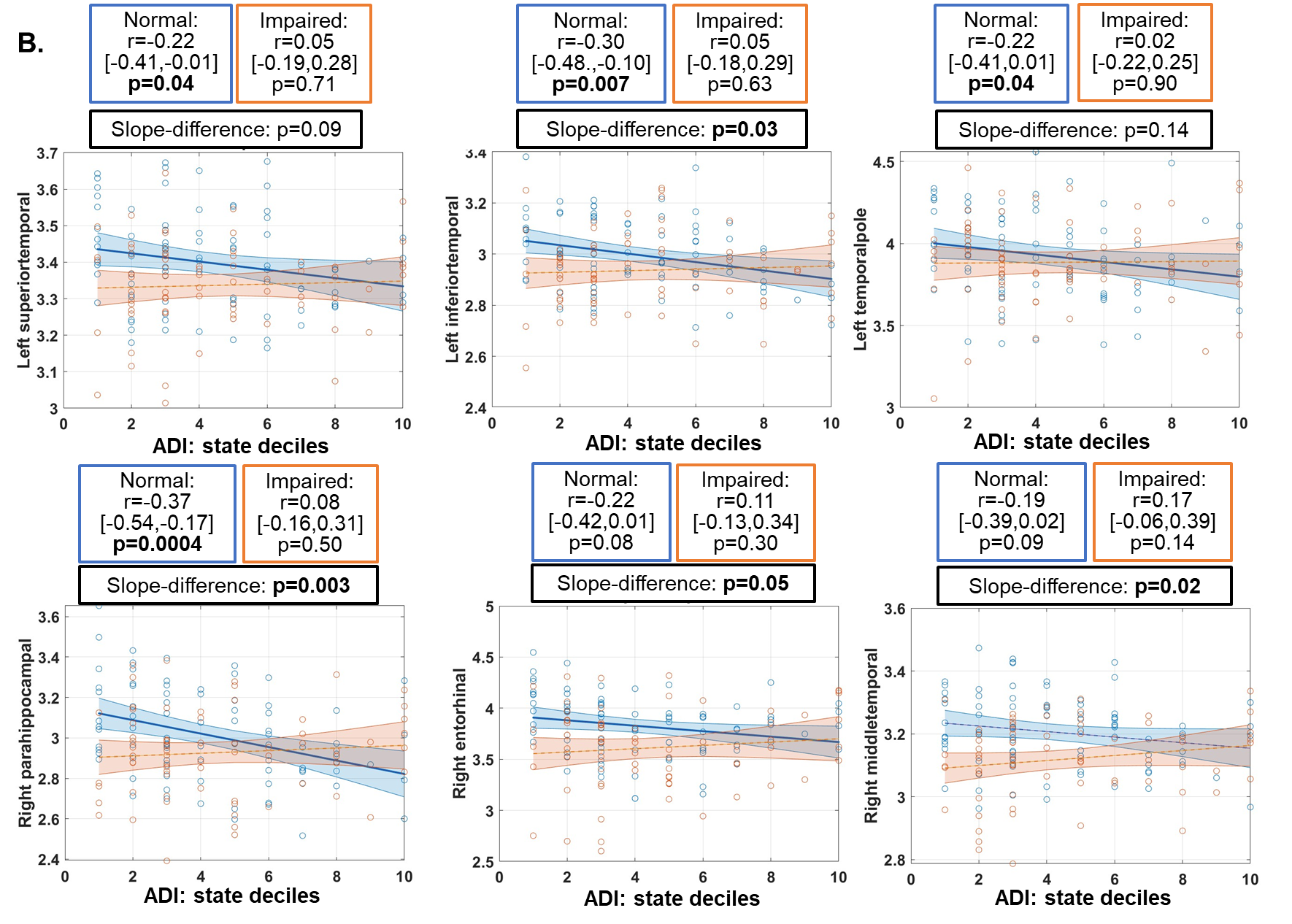


# Supplementary Fig. 6.

***(A).*** ANCOVA results on hippocampal volume. ***(B).*** Relationships between hippocampal volume and neighborhood area deprivation index (ADI) in both normal (blue) and impaired (orange) participants. ADI ranked within Nevada was used. Hippocampal volume measures used in this post-hoc analysis and plotted here have been adjusted for age, sex, education and total intracranial volume in the ANCOVA model.


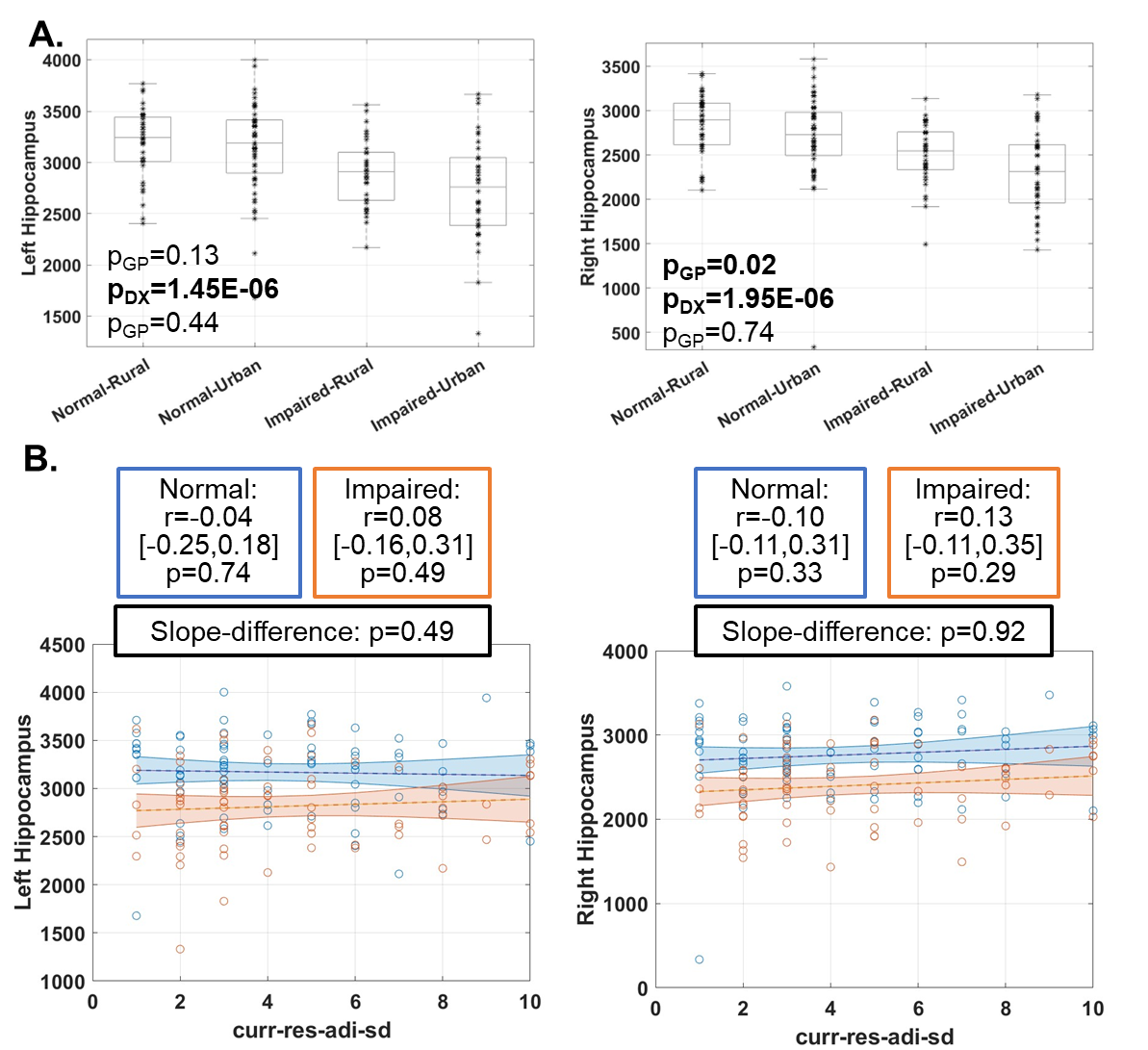


# Supplementary Fig. 7

**ANCOVA results after including ethnicity as additional covariates (cov). *(A)*** Significance levels (false discovery rate corrected *p-values (p_FDR_)*) in the main model and models with additional covariates. ***(B)*** Post-hoc effect-size (Cohen’s d) of residency effect (rural – urban) in all participants. ***(C)*** Cohen’s d of residency effect (rural – urban) in clinically normal (left) and impaired (right) participants.


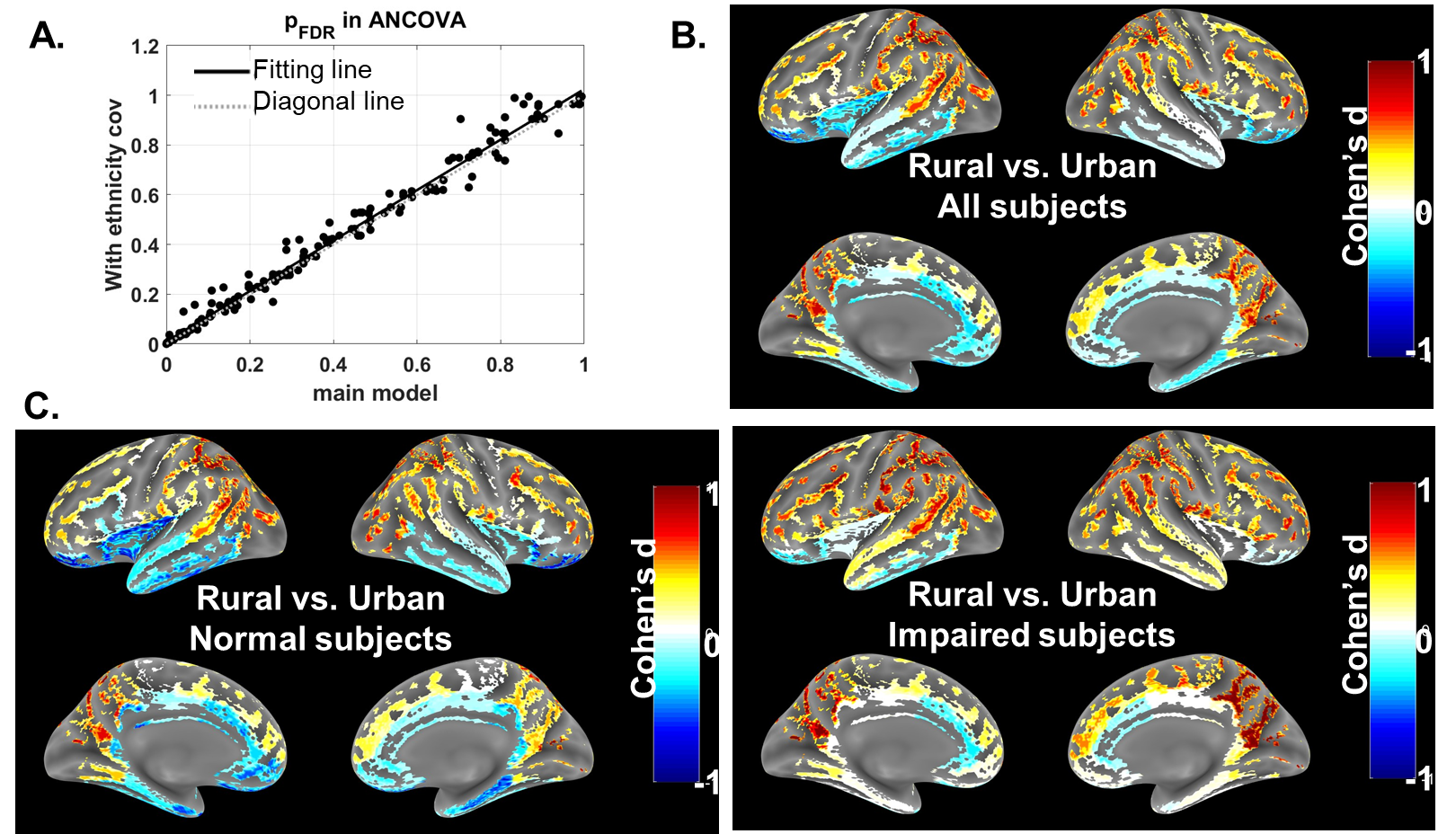


# Supplementary Fig. 8

**Sex-stratified analyses.** (A) Pearson’s correlation values (r) between sex-stratified analysis and main analysis. 95% confidence intervals are listed in square brackets. The observed effect-sizes for rural-urban differences and normal-impaired differences in cortical thickness measures (3^rd^ and 4^th^ rows) from all participants were highly correlated with those in separated male (2^nd^ column) and female participants (3^rd^ column), respectively. The reduced sample sizes in sex-stratified analyses might lead to the reduced correlation values among statistical significances in ANCOVA (2^nd^ row).

(B) Post-hoc effect-size (Cohen’s d) of residency effect (rural – urban) in all (2^nd^ column), normal (3^rd^ column) and impaired participants (4^th^ column) in main analyses (2^nd^ row) and sex-stratified analyses (3^rd^ and 4^th^ rows).

References:

1. USDA ERS - Rural-Urban Commuting Area Codes. Accessed December 26, 2023. https://www.ers.usda.gov/data-products/rural-urban-commuting-area-codes/

2. Health Resources & Services Administration. https://www.hrsa.gov/rural-health/about-us/what-is-rural#:~:text=We%20can%20identify%20rural%20census,codes%204%2D10%20as%20rural.

3. Kind AJH, Buckingham WR. Making Neighborhood-Disadvantage Metrics Accessible — The Neighborhood Atlas. *New England Journal of Medicine*. 2018;378(26):2456-2458. doi:10.1056/NEJMP1802313/SUPPL_FILE/NEJMP1802313_DISCLOSURES.PDF

4. University of Wisconsin School of Medicine and Public Health. Area Deprivation Index 2021. Downloaded from https://www.neighborhoodatlas.medicine.wisc.edu/ 2023.

5. Mora J, Krepline AN, Aldakkak M, et al. Adjuvant therapy rates and overall survival in patients with localized pancreatic cancer from high Area Deprivation Index neighborhoods. *The American Journal of Surgery*. 2021;222(1):10-17. doi:10.1016/J.AMJSURG.2020.12.001

6. Rangachari P, Govindarajan A, Mehta R, Seehusen D, Rethemeyer RK. The relationship between Social Determinants of Health (SDoH) and death from cardiovascular disease or opioid use in counties across the United States (2009–2018). *BMC Public Health*. 2022;22(1):1-19. doi:10.1186/S12889-022-12653-8/TABLES/5

7. Nevada Rural and Frontier Health Data Book - 10th Edition.

8. Fischl B. FreeSurfer. *Neuroimage*. 2012;62(2):774-781. doi:10.1016/j.neuroimage.2012.01.021

9. Desikan RS, Ségonne F, Fischl B, et al. An automated labeling system for subdividing the human cerebral cortex on MRI scans into gyral based regions of interest. *Neuroimage*. 2006;31(3):968-980. doi:10.1016/j.neuroimage.2006.01.021
